# A Mixed-Methods Study of How a Critical Race Theory-Informed Undergraduate Research Experience Program Provides Equitable Support for Aspiring Graduate Students

**DOI:** 10.3390/educsci14030334

**Published:** 2024-03-21

**Authors:** Frank Fernandez, Sarah Mason, Shannon Sharp, Gabriela Chavira, Crist S. Khachikian, Patchareeya Kwan, Carrie Saetermoe

**Affiliations:** 1 College of Education, University of Florida, Gainesville, FL 32611, USA; 2 Center for Research Evaluation, University of Mississippi, Oxford, MS 38655, USA; 3 Department of Psychology, California State University Northridge, Northridge, CA 91330, USA; 4 Department of Civil Engineering and Construction Management, California State University Northridge, Northridge, CA 91330, USA; 5 Department of Health Sciences, California State University Northridge, Northridge, CA 91330, USA

**Keywords:** survey data, qualitative, oral history, STEM, biomedicine

## Abstract

Numerous studies document the benefits of participating in undergraduate research experiences (UREs), including greater odds of enrolling in graduate school. However, there is a lack of understanding about *how* UREs support student success. This study examines survey and interview data from a multi-year program evaluation of a National Institutes of Health-funded biomedical training program to consider whether and how participating in a URE fosters students’ sense of belonging, which is an important predictor of retention and graduation. Analyzing the quantitative survey data revealed that participating in the URE was positively associated with a sense of belonging even after controlling for students’ background characteristics, including gender, race or ethnicity, first-generation status, commuting burden, and age. Additionally, there was a positive relationship between a sense of belonging and odds of applying to graduate school. Path analysis suggests that the URE has an indirect relationship with applying to a graduate program that operates through the URE’s direct relationship with sense of belonging. Interview data offered insights into how the URE supported an increased sense of belonging. Specifically, we found that the URE fostered a sense of belonging when (1) faculty research mentors develop authentic, personal, and caring relationships with mentees, (2) the URE program welcomes, cultivates, and supports women and racially diverse students, and (3) the URE is embedded within a university environment that allows for faculty and peer engagement.

## 1. Introduction

The National Science Board [1], National Science Foundation [2], and National Institutes of Health [3] have called for increasing diversity at all stages of the science, technology, engineering, and mathematics (STEM) and biomedical workforce, including in graduate education. Despite a chorus of calls for broadening the pipeline for preparing the nation’s scientific workforce, enrollment of undergraduate students of color declined disproportionately during the COVID-19 pandemic. According to the National Student Clearinghouse [4], enrollment of Black students at public four-year universities declined by 4.8% in Fall 2022 compared to Fall 2021. Hispanic and Asian enrollments at public four-year universities also declined during the same period, although to a lesser extent (1.2% and 1.0%, respectively). When underrepresented students of color do enroll in college, they have substantially lower retention rates (re-enrolling at the same institution one year after initial enrollment) than White students. Only 66% of Black students re-enrolled for a second year at the same public four-year university, compared to 78.5% of White and 87% of Asian students [5]. When students do not complete undergraduate degrees, they cannot pursue graduate degrees that prepare them to generate new knowledge, become faculty, or mentor future racially minoritized undergraduate students.

One reason underrepresented students of color may not re-enroll in college is that they often experience hostile or unwelcoming climates—that is, they encounter racism or discrimination—in higher education [6]. A substantial body of research demonstrates that students of color, and in particular women of color, find STEM courses and programs to be particularly problematic [7,8]. One phenomenological study of three Black women in STEM found their experiences were punctuated by “racial and gender discrimination, isolation, marginalization, and alienation” [9] (p. 202). Another phenomenological study relied on interviews with 17 Latinas and found that they felt skepticism, self-doubt, and isolation. Though prior literature has suggested that participating in STEM identity-based organizations can help support students, the Latinas shared that “an important reason behind their decision not to be involved with STEM identity-based organizations” was their “lack of sense of belonging with their STEM peers” [10] (p. 449). In other words, because Latinas felt like they had experienced sexism from peers in their STEM classes, they were reluctant to socialize with those same peers out of class. According to these Latinas, support systems that are tailored to retaining specific groups of students will have difficulty attracting students who do not feel like they belong in the first place.

The purpose of this paper is to examine whether participating in a biomedically focused undergraduate research experience (URE) program can help support a sense of belonging in college and whether sense of belonging relates to the odds of applying to graduate school. Since the late 1990s, UREs have become increasingly common across U.S. higher education [11]. Research shows that there are a number of short- and long-term benefits associated with participating in UREs, including enrolling in a graduate program [12–16]. Despite documenting many positive outcomes associated with UREs, there is an incomplete understanding of the mechanisms by which they support student success.

Astin’s theory of student involvement [17,18] suggests that the way students are involved in their education may be as influential as curricular content or skills. In other words, Astin posits that when students are more attached or committed to their university, they may be more involved and do better across a range of outcomes. Drawing on Astin’s theory of student involvement, we seek to examine whether a large, federally funded URE informed by critical race theory (CRT) may foster an increased sense of belonging among participating students. Specifically, this URE was informed by Sólorzano et al.’s articulation of the five tenets of CRT, including the “most basic premise that race and racism are defining characteristics of American society” [19] (p. 274). Additionally, Sólorzano and colleagues explain that CRT holds that “color blindness and race neutrality act as a camouflage for the self-interest, power, and privilege of dominant groups in American society”, that “CRT has a fundamental commitment to a social justice agenda”, that “the experiential knowledge of people of color is legitimate and critical to understanding racial subordination”, and, finally, that it is important to consider “race and racism in both a historical and a contemporary context using interdisciplinary methods” [19] (p. 275). The URE aligned programming, mentorship, and instruction with these tenets to address power dynamics in STEM disciplines, in higher education, and in society [20].

Sense of belonging is defined as a “psychological sense that one is a valued member of the college community” [21] (p. 804). Though it is not a concrete outcome like re-enrolling or graduating from a university, it is “a key antecedent to retention in college” [22] (p. 1) and has been found to have “direct effects on institutional commitment and indirect effects on intentions to persist and actual persistence behavior” [23] (p. 649). Because there is a lot of variation in how UREs are designed [24] and questions about whether UREs are more beneficial than research-intensive courses [25], it is important to better understand whether and how a URE may foster sense of belonging that could facilitate better affective, skill, and academic outcomes.

The findings presented in this paper are part of a larger, five-year project that uses a mixed-methods research design to consider how one URE program uniquely supports sense of belonging among student participants relative to non-URE students at the same university—and how sense of belonging is an important predictor of applying to pursue graduate education. By using survey data and statistical methods, we are able to test for differences in sense of belonging among the two groups of students. We also test the relationship between sense of belonging and odds of applying to graduate school. Then we use path analysis to test direct and indirect effects among URE participation, sense of belonging, and applying to attend graduate school. We complement our quantitative results with interview data to offer examples of how students remember and narrate their involvement with the URE program and how their experiences fostered or inhibited their sense of belonging. Our findings complement prior literature and offer implications for how UREs can foster sense of belonging, which may facilitate other benefits to students, their university, and society.

## 2. Sense of Belonging in Higher Education

There is a substantial body of research that examines relationships between student experiences and sense of belonging. Bollen and Hoyle were the first to operationalize sense of belonging by developing a measure that asked students how strongly they felt “a sense of belonging”, whether they felt that they were “a member of the community”, and whether they “see [themselves] as part of the community” [26] (p. 485). Many studies have used the concept of sense of belonging to examine data from students in the same stage of college. For example, Freeman et al. [27] examined sense of belonging among first-year students. They found that students with high sense of belonging described interacting with faculty who they perceived as caring deeply about whether students were learning (something the authors referred to as *pedagogical caring*) and who were approachable and facilitated student participation. Johnson et al. [28] also emphasized that experiences related to living on campus can influence sense of belonging. Conversely, living away from campus creates challenges for commuters who have less time and occasion to develop relationships with faculty or be involved with peers on campus [29].

Other studies suggest that it is important to consider how students develop sense of belonging as a longitudinal process. For instance, De Sisto et al. focused on sense of belonging in the second year of college. They argued that after the initial transition to college as first-years, “the novelty of starting their degree has worn off, but they have not completed enough of it to feel part of their academic field” [30] (p. 1729). Hurtado and Carter [31] showed that it is important to analyze data for students at multiple stages of their undergraduate careers and to account for how long students were enrolled in college because sense of belonging in one year can influence sense of belonging in subsequent years. York and Fernandez [32] also found that it is important to consider how long students have been at a particular campus because their analysis suggested there can be a curvilinear relationship between campus experiences and sense of belonging (e.g., initial gains can be followed by a loss, which ultimately becomes positive again).

In addition to temporal variations in sense of belonging in college, one study showed that first-year students’ sense of belonging varied substantially by race, with students of color having lower sense of belonging than White students [28]. Subsequent studies have examined specific subgroups, such as Hispanic students [33] and Black students [34]. Like Freeman et al. [27], Nuñez found that “faculty interest in the student’s development also appears to have a strong, direct, and positive effect on sense of belonging” [33] (p. 56) among Hispanic students. Consistent with Nuñez’s study of Hispanic students [33], Strayhorn [34] found that among Black students, interaction with diverse others was positively associated with sense of belonging.

A few studies have begun to examine sense of belonging among students in the biomedical sciences or who participate in UREs. Hurtado et al. [35] examined survey data from first-year students who aspired to work in biomedical and behavioral sciences; they found that sense of belonging was positively related to favorable academic and social experiences but negatively influenced by concerns about paying for college. When it comes to working in a research setting, Vannier et al. examined students’ experiences in a biomedical sciences summer internship program. They found that among the students of color in the program, some described “the anxiety some interns experienced” when they were the only members of underrepresented groups in their labs”, although the authors did not explicitly link that anxiety to “negatively impact[ing] interns’ sense of belonging” [36] (p. 9). Other scholars have called for new research to examine students’ sense of belonging “during and after” participating in an URE [37] (p. 20). It is worth noting that not all students participate in research with faculty with equal frequency. For example, first-generation college students have lower odds of involvement in faculty-led research [38].

## 3. Methods

The work presented here is part of a five-year program evaluation of California State University, Northridge’s (CSUN) Building Infrastructure Leading to Diversity (BUILD) Promoting Opportunities for Diversity in Education and Research (PODER). BUILD PODER offers a URE program that focuses on training students for graduate school and careers in the biomedical sciences. It was first launched in 2014 with support from the National Institutes of Health (NIH). BUILD PODER is part of the NIH’s Diversity Program Consortium, which includes nine additional sites for training university students for careers in biomedical research. BUILD PODER contributes to the consortium’s National Research Mentoring Network and the Coordination and Evaluation Center to coordinate activities, evaluation strategies, and identify best practices for UREs.

The URE evaluation plan aimed to examine outcomes for students who participated in the NIH-funded URE at CSUN by comparing them to students in similar majors who did not participate in the URE experience. Students were eligible to apply to participate in the URE if they had an undergraduate GPA of 3.0 or higher and were enrolled in a baccalaureate degree-granting program in a biomedical-related field at CSUN (or with the intention of transferring to complete a degree at CSUN). BUILD PODER is the first URE guided by CRT frameworks that prioritized improving diversity in biomedical research both through whom it admitted and in the design of its activities and programming, including the selection and training of faculty mentors in being culturally response. The URE provided students with tuition remission, monthly stipends, conference travel, and research funds to work on faculty mentored research projects. More importantly, the students received CRT-informed curriculum to reveal the “hidden curriculum” of academia and provide wholistic support for students from underrepresented groups to increase persistence and graduation rates and the likelihood of enrolling in biomedical-related postgraduate training. See [20,39] for more information about how the URE was developed.

The evaluation plan used an explanatory mixed-methods approach [40–42] to (a) test whether there is a relationship between participating in the URE program and sense of belonging, (b) test whether there is a relationship between sense of belonging and applying to graduate school, and (c) understand how the URE may foster—or fail to foster—sense of belonging. Creswell et al. [41] defined mixed methods as a distinct research design rather than a mere fusion of quantitative and qualitative designs. They argued that mixed-methods designs must consider whether quantitative and qualitative data are collected concurrently or sequentially, whether each type of data is given equal priority, and whether data are integrated at multiple stages of the research project (e.g., data collection, data analysis, and interpretation of findings). This five-year project followed a mixed-methods design that relied on quantitative (survey) and qualitative (interview) data being collected, analyzed, and interpreted concurrently with equal priority. In this way, “quantitative methods can estimate the statistical significance and relative size of relationships” among URE participation, sense of belonging, and odds of applying to graduate school, even as qualitative methods are useful for understanding how a URE can facilitate the path to graduate school by helping to “uncover processes, illuminate experiences, and describe their contextual significance” [42] (p. 194). The study design was approved by the Institutional Review Board at the University of Mississippi with a Reliance Agreement with California State University, Northridge.

## 4. Survey Data Collection

The research team used Qualtrics to email an expression-of-interest form to students in the URE and like-major students not in the URE. The initial interest form sought to collect permanent contact information and background information (e.g., age, gender, first-generation status, race/ethnicity) for URE participants. The background characteristics were needed, in part, to identify and create a comparison group of non-URE students. Following a matched comparison quasi-experimental design [43], the research team sought to collect enough information from a pool of students to create a comparison group that was similar in terms of demographic characteristics to the URE students. The research team distributed a separate expression-of-interest form to more than 2500 CSUN students who were previously identified as part of a comparison group for an NIH consortium-wide evaluation. As a selection criterion for the comparison group, students had to meet the URE eligibility criteria (3.0 GPA, enrolled in a degree-granting program). To further expand the pool of students who could be selected for the comparison group, the expression of interest form was also distributed to undergraduate students at CSUN who were enrolled in one of four colleges from which students were eligible to participate in the URE.

After emailing the NIH consortium comparison group and the new list of CSUN students, the research team received more than 1000 responses to the expression of interest form. The research team used data from the form to create a comparison group to the URE students by matching on background characteristics, including age, gender, first-generation status, and race/ethnicity. Specifically, the research team used a nearest-neighbor propensity score matching approach [44]. T-tests demonstrated that the matched comparison group resembled the treated group of URE students without any statistically significant differences between the two groups.

In each year of the evaluation, the research team distributed the expression-of-interest form to new URE students (and used matching procedures for non-URE students) to expand the panel. After collecting information from the expression-of-interest form, the research team administered an online survey to URE participants and those who were in the matched comparison group. As new students joined the survey panel, their data was incorporated into the program evaluation (see Table 1). Survey completers received an online gift card that increased in value by USD 5 increments for each year they participated (i.e., USD 25 in the first year, USD 30 in the second year. Because the panel is unbalanced (i.e., the research team does not have data for all years for all students), we analyzed the most recent cross-section of current students and alumni who completed the survey in spring 2023 (*n* = 278). Demographics for the 2023 survey completers are included in Table 2.

In addition to updating background or demographic data, the annual survey included five-point Likert-scale items from previously validated measures [45,46], which we used to create a single, summative scale score as a proxy for *Sense of Belonging*. The five items are: (a) “I feel like I belong at my university/community college”. (b) “At my university/community college, I feel secure”. (c) “At my university/community college, people look out for each other”. (d) “I am involved in my university/community college community”. and (e) “I am satisfied with the social support I receive from my university/community college community”. The scales [45,46] share the first item: “I feel like I belong at my university”. The items were modified to the past tense for surveys that went to alumni (e.g., “I felt like I belonged…”).

We previously analyzed the scales [45,46] separately and found that they had comparable reliability with this panel of respondents when compared to the original validation studies. We also showed that they were each positively related to a measure of CRT-informed mentoring, which was a component of this URE [47]. For this sample, combining the items [45,46] into a single scale resulted in higher scale reliability than using either set of items separately (Cronbach’s α = 0.81 for current undergraduates, Cronbach’s α = 0.84 for alumni, and Cronbach’s α = 0.83 for the pooled sample of current undergraduates and alumni). Additional *Sense of Belonging* descriptive statistics from the 2023 annual survey are included in Table 3. Finally, the survey asked respondents whether they had applied to graduate school (1 = yes, applied to at least one graduate program; 0 = no). Fifteen current undergraduates and 37 alumni reported they had applied to graduate school at the time they completed the survey.

## 5. Interview Data Collection

The qualitative data were collected via in-depth oral history interviews [48]. Oral history emphasizes “personal narrative as a valid articulation of individual and collective experience with the social, political, and cultural worlds of education” (Errante, 2000, p. 16). Narratives go beyond merely recounting prior experiences or sequences of events; they “declare narrators’ alignments with certain “in” individuals, groups, ideas, and symbols onto which they externalize their most valued, positive, and pride-inducing qualities” [48] (pp. 16–17). Prior literature emphasizes that students develop a greater sense of belonging when they feel and see themselves as members of a community. Therefore, the oral history approach to interview data collection aligned with the research team’s aim of understanding how URE students narrate their relations to faculty mentors, research groups, as well as the broader campus.

When participants completed expression-of-interest forms (including both those in the URE and in the comparison group), they were invited to indicate whether they were willing to participate in the oral history part of the study. They were informed that interviews would ask them to remember and narrate their experiences in college, and they were asked to consent to being contacted over multiple years and to commit to participating in approximately one-to-two-hour interviews each summer for the duration of the study. Narrators (i.e., interviewees) were invited to schedule interviews by e-mail, and follow-up phone calls were used as a last attempt to contact when narrators did not respond after three e-mail attempts.

**Figure 1.**
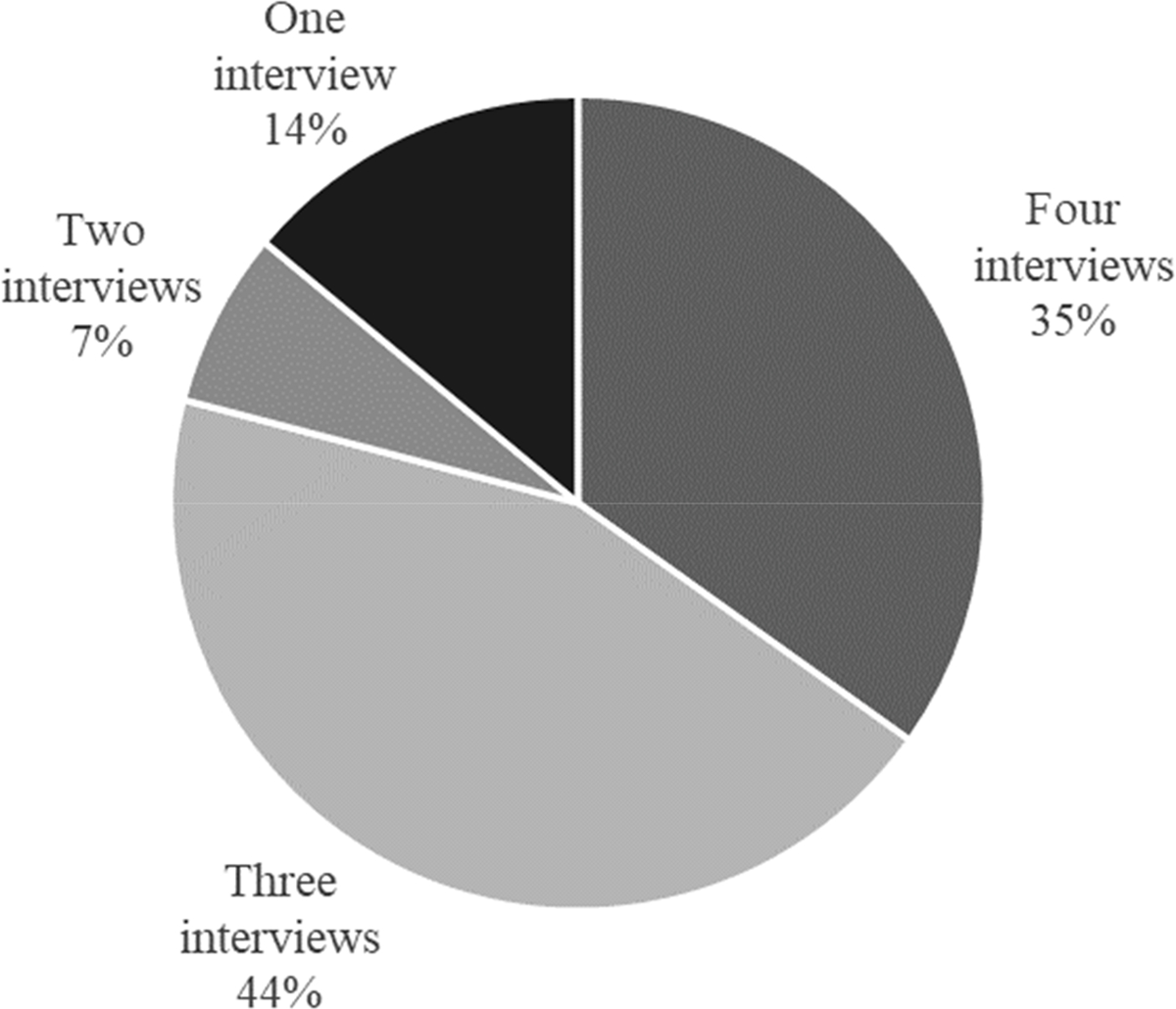
Summary of narrator participation throughout study.

In 2020, 2021, and 2022, the research team collected 28, 36, and 36 oral histories, respectively. By 2023, the team only collected 29 oral histories, which was due, in part, to attrition as alumni entered graduate school or careers and became less responsive or changed contact information. Narrators completed different numbers of interviews depending on the year they entered the panel and their availability across years (see Table 4). Fifteen narrators completed four interviews, 19 narrators completed three interviews, three narrators completed two interviews, and six narrators only completed one interview (see Figure 1). All interviews were conducted and recorded through Zoom. Initial transcripts were prepared by rev.com, and interviewers checked the transcripts and used audio recordings to identify and correct omissions or inaccuracies. Narrators were offered USD 25 online gift cards (increasing in value with each year of participation) to either Amazon or Target at the end of each interview.

## 6. Survey Data Analysis

First, *t*-tests were used to determine whether the differences in means for the *Sense of Belonging* scale were significantly different between students participating in the URE and the comparison group. Then, the research team used ordinary least squares estimation to examine whether the relationship between URE participation and *Sense of Belonging* was statistically significant after controlling for gender [10], race [28], first-generation status [38], commuting burden [29], and time in/out of college [31]. We estimated three separate models to test whether our findings were robust. The first model was limited to analyzing data for survey completers who were still in college (i.e., current undergraduate students). The second model was limited to analyzing data for survey completers who had graduated from college (i.e., alumni). The third model included both survey completers who were both current undergraduate students and alumni. We reported results with standardized beta coefficients to interpret findings in terms of standard deviations of the *Sense of Belonging* outcome variable.

After examining whether URE participation was related to sense of belonging, we used logistic regression to examine relationships among URE participation, sense of belonging, and odds of applying to graduate school. Below, we present findings for the pooled sample of current undergraduates and alumni. As a robustness check, we repeated the analysis for alumni only and found similar results. In addition to logistic regression, we used path analysis to parse direct and indirect relationships among these three key variables (URE participation, sense of belonging, applying to graduate school). We acknowledge the limitations of using cross-sectional data and correlational methods for all our quantitative analysis and caution readers against inferring causality from the findings. Nevertheless, the survey data analysis complements the qualitative data (discussed below) to provide a parallel understanding of how students can benefit from a URE that is informed by CRT.

## 7. Qualitative Data Analysis

Qualitative data analysis has long been seen as an important element of effective evaluation research, whereby the evaluator serves as an instrument of both data collection and analysis to discover “idiosyncrasies rather than norms” and understand “that which is unique, atypical, different, idiographic, individualistic” [49] (p. 129). In this context, the oral history interview data were systematically and iteratively analyzed each year. The research team analyzed interview transcripts following Saldaña’s [50] coding process, which involves multiple rounds of coding.

First, researchers generated initial codes from the transcripts using in vivo coding or the narrators’ own words. The research team reviewed the transcript data, created an inventory of codes, jointly reviewed work to achieve *intercoder agreement* or *interpretive convergence*, and sought to identify patterns among the codes within and across interview transcripts [50]. Then, using an axial coding approach, codes were grouped into categories to better understand how individuals describe phenomena. Finally, categories were aggregated into themes that reflected how the URE fostered—or inhibited—narrators’ sense of belonging [50]. Quotes were selected from narrators’ transcripts to provide a “thick description” of how they remembered the URE as fostering sense of belonging or having exclusionary experiences [51].

## 8. Results

**Figure 2.**
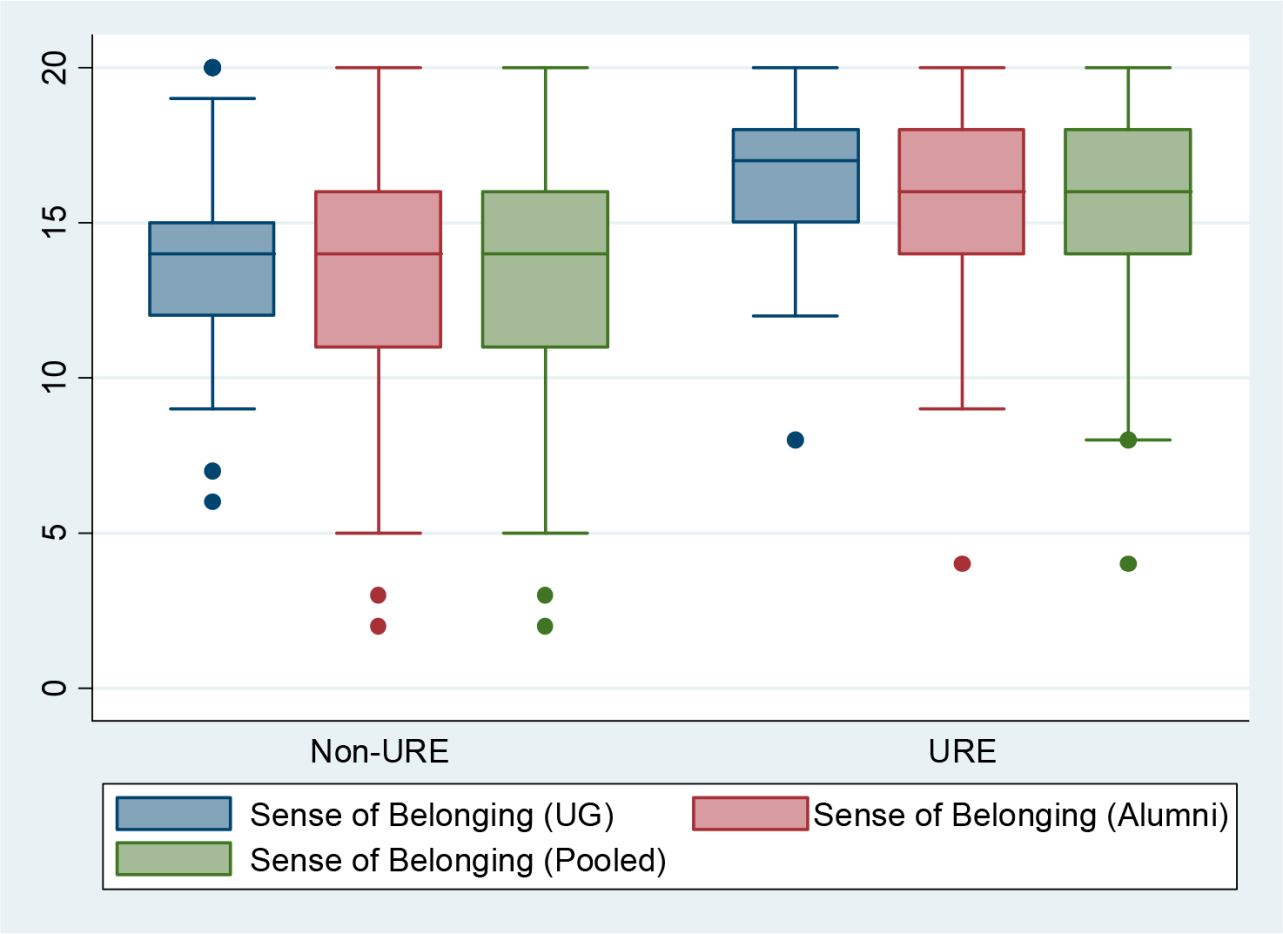
Box plot of sense of belonging scale scores grouped by participation in an undergraduate research experience (URE) program. Note: The horizontal lines within the boxes represent medians, and the shaded areas surrounding the medians include the 25th (lower) and 75th percentile (upper).

Through the mixed-methods research design, quantitative data were used to test whether there was a substantive difference in outcomes between students who participated in a URE (BUILD PODER) and a comparison group of similar students. Results from *t*-tests showed there was a statistically significant difference (*p* < 0.01) in mean *Sense of Belonging* scale scores between current undergraduates in the URE (*M* = 15.94; *SD* = 3.13) and current undergraduates not in the URE (*M* = 13.73; *SD* = 3.04). Similarly, among alumni reflecting on their connection to CSUN, those who had participated in the URE had a higher *Sense of Belonging* (*M* = 15.55; *SD* = 3.58, *p* < 0.01) than those who had not participated in the URE (*M* = 13.50; *SD* = 3.75). For the pooled sample of both current undergraduates and alumni, those who had an affiliation with the URE had a higher *Sense of Belonging* (*M* = 15.68; *SD* = 3.35, *p* < 0.001) than those who did not (*M* = 13.56; *SD* = 3.55). Figure 2 displays the bivariate relationship between participation in the URE and *Sense of Belonging*.

Multivariate analyses confirmed that participating in the URE was positively related to *Sense of Belonging.* After controlling for background characteristics, time in college, and time in the study, current undergraduates’ participation in the URE was positively associated with an approximately one-third standard deviation increase in *Sense of Belonging* (*β* = 0.34, *p* < 0.1). Among those who had graduated and reflected on how strongly they felt like they belonged in college, participating in the URE was related to having one-fifth of a standard deviation higher *Sense of Belonging* (*β* = 0.20, *p* < 0.01). In the model that included both current undergraduates and alumni, participating in the URE was associated with an increase in the *Sense of Belonging* scale score (*β* = 0.23, *p* < 0.001). See Table 5 for additional results.

Next, logistic regression indicated that *Sense of Belonging* was positively and statistically related to respondents’ odds of applying to graduate school. For this step, we present findings using a standardized version of the *Sense of Belonging* variable for ease of interpretation. Results indicated that a one-standard deviation increase in *Sense of Belonging* related to being approximately 1.8 times more likely to apply to graduate school. Otherwise stated, someone who had Sense of Belonging that was one standard deviation higher than the mean had about 80% higher odds of applying to graduate school. See Table 6.

**Figure 3.**
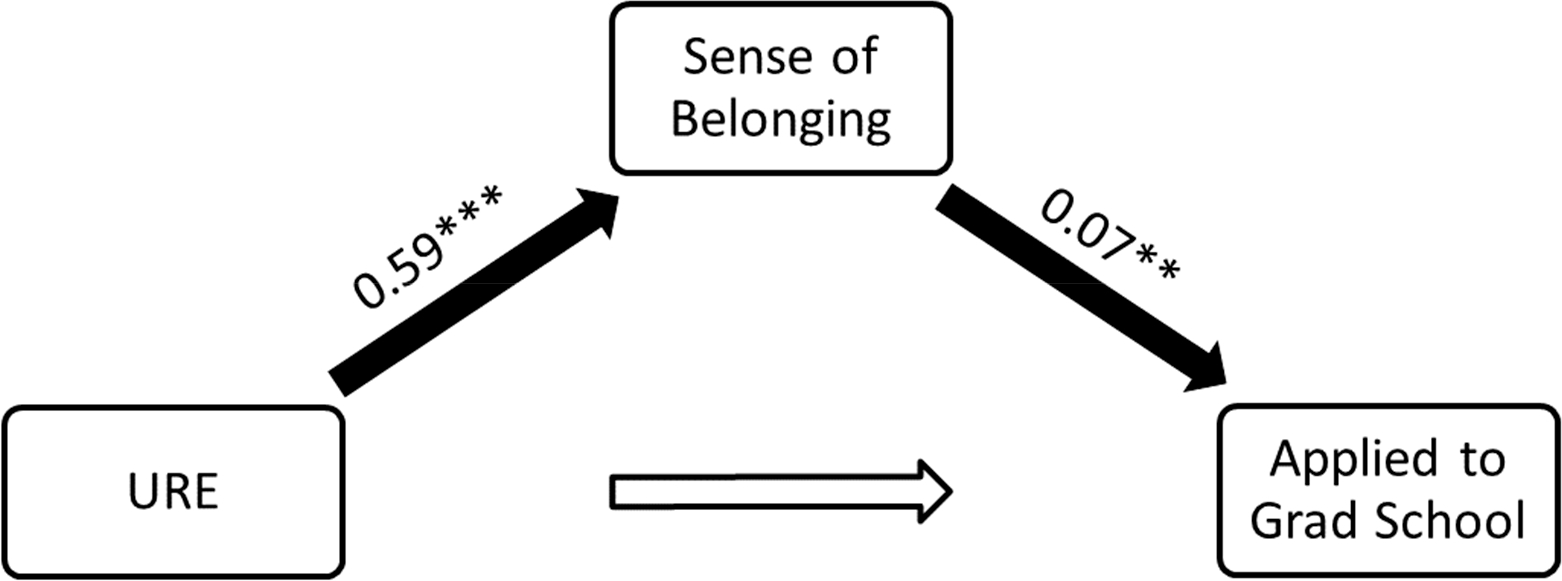
Path analysis displaying indirect relationship of URE participation on odds of applying to graduate school. Note: *n* = 277, *R*^2^ = 0.04, ** *p* < 0.01 *** *p* < 0.001.

Note that in Table 6, the parameter estimate for participating in the URE was not statistically significant. Building on the results in Tables 5 and 6, path analysis indicated that URE participation had a positive, statistically significant direct relationship with the standardized *Sense of Belonging* variable but not with the outcome for having applied to graduate school. *Sense of Belonging*, however, did have a positive, statistically significant relationship with applying to graduate school. See Figure 3.

After confirming that URE was positively associated with statistically significant differences in *Sense of Belonging* scores and that *Sense of Belonging* positively related to having applied to graduate school, the oral history interview data contributed to the mixed-method design by providing insights about how the URE fostered belonging among participating students. The research team identified 10 categories of codes in the transcripts. From those categories, the researchers arrived at three themes: (1) Faculty communicate to students that they belong; (2) by establishing program composition, UREs can create local communities of belonging; (3) the university environment is congruent with the URE’s environment and supports positive involvement in the campus community. See Table 7 for a summary of how the three themes were developed from the ten categories. All the quotes presented below come from URE participants.

### 8.1. Theme 1: Faculty Communicate to Students That They Belong

URE participants narrated memories of feeling like they belonged when they felt they had personal connections with their faculty research mentors. Although they did not fault other university faculty, they described a distinction between the types of relationships they had with URE faculty and the non-URE faculty with whom they completed coursework. For instance, one student stated:

The first time that I spoke to her, she was very lovely and very… caring… There’s only [a few] full-time professors in our department… and they didn’t really care how you were feeling as a student, like if you were struggling… They were very not caring. I don’t know what’s the opposite of caring, not caring? Just it seemed like they didn’t really care sometimes for the students.

A different student shared something similar, which suggests that the prior quote was representative of how multiple students benefitted from relationships with faculty that occurred through the URE.

I think BUILD really wants to see you succeed no matter. And you know that they’re all busy, but you also know that they’ll put time aside for you and they’re very open for not just questions about class or requirements and stuff, but they’re more than happy to talk to you about even your personal life. If you really needed someone to go to, there’s someone in the staff that you can confide in. … And I guess you don’t get that with your [non-URE] professors…being in BUILD you have this sense of ‘this is a person’ and not only are they faculty but they’re also kind of like my friend, like a mentor too.

A third example supports this perspective: “I feel like they personally know me and so they personally care about…my academic welfare, doing well in school and just me overall. My other professors…I guess they don’t know me personally, so they don’t really care that much”.

When faculty members expressed interest in students as holistic people, students felt seen and understood. The students believed that faculty were interested in learning about them and their career goals. One student described getting to better know her faculty mentor and feeling understood.

I’m very, very fortunate and grateful to have a mentor that I can relate to in a lot of ways. And I think the experiential knowledge of being a woman, I think, has a huge impact on that for sure…

Being able to see how I can relate to her because of the way that she went through school or the difficulties that she had, and also just knowing that she can understand how it is being a woman or dealing with sexism or anything related to any of the challenges that she had in that respect … It’s really amazing because it’s being able to see that not only can a person, who is like me, get through this stuff, but that I can potentially become a helpful, amazing person like her.

Whereas some students described being recognized and understood by faculty, which may be seen as subtle forms of caring, other students explicitly described feeling like professors cared about them. One student stated: “I felt like all of the professors were super…they really cared”. Another student in the URE described being surprised by the level of caring and how that care connected addressing community needs through research.

I was like, “Wow! She cares about us, about the students”, and that’s the first time that I see that from one professor. … We share the same similar background, and then she was doing a lot of work with the Latino community. I was like, “We need more people like you. We need more people working in the Latino community” … so we can fill those gaps when it comes to health and economically.

When faculty mentors exhibit care and empathy, students become more open to the faculty’s efforts in pushing and motivating them to step out of their comfort zones and strive for self-improvement. In the narrative below, the student sees participation in conferences and internships as emerging from their mentor’s caring encouragement rather than as program requirements or deliverables.

She also nicely pushed me to do things, like, “Go to [a] conference and present. Go to this internship, apply for this”. She brought so many things. And I wouldn’t have done any of those things if it wasn’t for her. Every time I had to go to a conference, I didn’t really want to. Every time I applied for something, I was scared to go. But they all worked out. When I presented at conference, it was a great experience. … she encouraged me, like, “You have to do this to get prepared towards your PhD”. Yeah. I think that’s something I look back upon and find that’s been very impactful.

Taken together, these interview data suggest that one way the URE facilitates sense of belonging is by connecting students to faculty mentors who show authentic interest in their mentees, who develop personal connections with students, and who help those students feel cared for in the URE.

### 8.2. Theme 2: By Establishing Program Composition UREs Can Create Local Communities of Belonging

Multiple students characterized the diversity of the students and faculty (i.e., gender, race/ethnicity) who were involved in the URE as the norm and identified it as a strength of the URE. Students indicated that because there was so much diversity, people did not stand out as different and everyone fit in. For instance, one student noted: “I can just walk around and be who I am”. Other students described feeling like they belonged because they saw other people “like me”. Consider how one student described meeting her research mentor and the transition from guarded skepticism to shared cultural understanding.

When I met her, I was like, “I don’t know this lady, she hasn’t been my professor here”. I knew her because she was one of the department professors, but I talked to her, and she was lovely, and she cared about how I felt with my struggle… when I find out that she was Latina, that she grew up in (Location), her parents were from (Country), so she shared those two cultures very well, the American culture, but at the same time the (Country) culture.

When students referred to being around others with shared identities, they described feelings of comfort. They suggested they felt at home because they could rely on having at least a few people around them who understood where they were coming from (e.g., geographically, culturally). At a minimum, if the diversity of the URE does not facilitate sense of belonging, it helped them avoid feeling “out of place”. One student stated:

That environment became a family to me in the two or three years. So, not only do we see each other in our labs and support…but we’re hanging out. We have a lot of people that have already graduated, we still always make time for each other. So, I think I would usually go to them for support in sense of lab work or just personal.

When students did not feel isolated or “out of place”, the environment of the URE allowed them to reach out to others and become increasingly involved in faculty members’ research teams. One student described first getting to know people as members of a research lab, then feeling comfortable enough to ask for help with coursework, and eventually socializing with them outside the lab.

In the beginning, I wouldn’t have reached out to them for anything, but as I went on, … they would always make sure that they were there. So, “if you need anything, I’ll be there”, kind of thing. I would reach out to them a lot for that, and some of the master’s students were very, very proficient in subjects like A or B so, sometimes, I would go to them for tutoring too. … most of those people in that lab were very outgoing and outspoken. So, you didn’t have a chance to not socialize, if you weren’t being social, they were going to make you be social. So, it was a really, “you have to hang out with us” environment. … they want you to be friends, they want you to be comfortable.

Students described flourishing in the URE culture of diversity. They appreciated that they shared backgrounds and felt similar to others (faculty or students) in the URE. They also discussed how they became increasingly involved and connected to others in that environment. These dynamics offered validation and confirmed that, despite temporary self-doubt or failures, students could still belong in the URE and in higher education. One student declared that the URE helped her develop

Confidence, for sure. Confidence to be a researcher, confidence in myself as a woman, confidence to apply to graduate school. I guess I finally proved to me that I am smart, and even if you don’t necessarily get an A in everything… I didn’t expect that it was going to provide the emotional support that I have gotten, and so it’s been really, really, great to be honest. One of my favorite parts about the program is the emotional and wellness support that I have gotten. And I love the research aspect of it…But without the emotional support, I don’t think that I’d be able to be as confident and as good of a researcher and student as I am today.

The second theme complemented the first. In addition to pairing students with trained faculty mentors who can extend caring and build relationships, the URE also helped create localized research-oriented communities (within a broader university setting) where students felt like they belonged.

### 8.3. Theme 3: The University Environment Is Congruent with the URE’s Environment and Supports Positive Involvement in the Campus Community

In addition to relationships with individual faculty mentors and members of research groups or the URE cohort, narrators described feeling like they belonged when recalling the friendly and helpful interactions with people on campus. For narrators, this was connected to whether they felt there existed “people I can relate to” on campus. The concept of *relatedness* could be created through a shared experience or a common background. As in Theme 2, narrators acknowledged that when they shared a common background with people they interacted with, they were more likely to feel that those individuals would understand them better.

Students also felt a stronger sense of belonging when they described the broader campus as welcoming, examples of which include faculty- and student-initiated invitations to engage (e.g., in office hours or interest groups). CSUN is a large, urban, public university, and the students appreciated the wide variety of interest groups that are available to them. Multiple students noted that because there are so many different clubs or groups, practically every student could find an interest group that appealed to them.

### 8.4. Additional Finding: Experiences Inhibiting Sense of Belonging

Most students’ oral histories (within each year and across years) described positive experiences with the URE. However, they occasionally remembered negative or exclusionary experiences with URE faculty and with non-URE faculty at the university that did not support their sense of belonging. Some of these are described below to provide a contrast to the positive experiences outlined above.

Students had weaker feelings of belonging when they believed faculty were not approachable, which occurred when faculty members saw them as a burden or indicated they were frustrated by student questions. For instance, one student stated: “I’m pretty sure Dr. [A] doesn’t like me … every time she talks to me, it feels like she’s completely annoyed with me and frustrated that she has to even deal with me”. Another narrator stated: “Dr. [B] is my PI…She’s kind of scary… she has this ‘looking for things to be disappointed in’ kind of attitude”. Some students felt that faculty “obviously don’t care about teaching and only want to do their research”. Others were less likely to feel they belonged in the classroom when they felt they were wasting faculty members’ time (e.g., faculty were too busy for them). At times students took this personally, which led to the view that faculty members did not like the student (e.g., “I’m pretty sure X doesn’t like me”). Narrators suggested that when they saw faculty as not approachable, they disengaged from interactions with those faculty.

In more overt terms, some students described instances when faculty members explicitly put them (or other students) down. This was variably described as students being “berated” or “scolded”. For example, one student described a faculty member saying: “If you can’t understand this super basic easy stuff, you should probably just kill yourself now”. Another described faculty members telling them: “you should know that—why don’t you know that?!” in response to asking a content-related question. One student described an experience where she felt threatened by a faculty research mentor.

She said, “Your letter of rec, it’s going to be bad…” She was like, “I’m going to have to say in your letter that you’re unreliable”. She was being mean to me. I was like, “I’m the most quiet in the lab, the most easy-going. You don’t have to do this to me, of all people”. … She uses fear tactics. She scared me. I was 19, 20. But she scared me. … I felt like I was very insecure: “Am I really a scientist? Maybe I should just go to Master’s and just become a teacher, something that doesn’t require so much letters of rec and work”. So that really messed me up. … They say, “BUILD’s a family”, but she left me behind.

Narrators also outlined a broader set of experiences that inhibit developing sense of belonging. They included encounters that challenged their self-worth. For students from racially minoritized backgrounds, needing help was sometimes seen as an indication that critics were right, and they did not belong. For example, one student shared feeling that “If I need help, I’m proving those who think I shouldn’t be here right”. Others described lacking personal connections or not fitting in, particularly when groups or activities were large and impersonal. Finally, narrators pointed to encountering explicitly racist or sexist behavior as affecting their degree of comfort in their classes, programs, or university campus more broadly.

## 9. Discussion and Implications

As federal agencies and universities continue to invest in expanding opportunities for women and underrepresented students of color to pursue careers in STEM and biomedical research, they must overcome the hurdle that many women, students of color, and women of color, in particular, do not feel a sense of belonging in the sciences [6,9,10] and often have higher attrition rates than other students [7,8]. One common approach to supporting students in STEM and the biomedical sciences is to offer URE programs [11,35]. Multiple studies have shown that UREs positively influence a broad set of outcomes [12–16]. However, prior research tends to overlook *how* UREs support student success.

Universities have many options to choose from when designing or expanding UREs [24,25]. Even when well intentioned, prior research documents that otherwise successful programs can leave students feeling anxious about being the only student of color in the research team [36]. Because prior research identifies students’ sense of belonging as an important prerequisite to persistence and intent to graduate, the purpose of this paper was to consider whether and how a URE (i.e., BUILD PODER at CSUN) fostered sense of belonging as an intermediate outcome in students’ development as scholars.

This paper presents a mixed-methods case study of one URE that was framed by CRT, integrated CRT into student and mentor trainings, and focused on serving students from underrepresented groups in the biomedical sciences (including women, students of color, and women of color). Although legislators in many states are working toward eliminating CRT from higher education [52], this study shows that, to the extent that CRT was embedded in this URE, CRT was not incompatible with efforts to promote STEM research and pathways to STEM graduate education. URE participants’ quotes suggest that CRT enhanced—rather than detracted from—their preparation to do research and pursue graduate degrees.

Results from statistical analyses of survey data confirmed that students in the URE had higher sense of belonging than students in the comparison group who did not participate in the URE. We also showed that sense of belonging predicted applying to graduate school. Following an oral history approach [48], we found that those who participated in the URE offered specific examples of how the program facilitated academic and social engagement, which are two key types of involvement in college [17,18] and important contributors to sense of belonging [31]. The research team’s analyses of interview transcripts resulted in three themes that described how the URE fostered sense of belonging: (1) Faculty communicate to students that they belong; (2) by establishing program composition, UREs can create local communities of belonging; and (3) the university environment is congruent with the URE’s environment and supports positive involvement in the campus community.

These findings complement prior work and offer transferrable implications for improving existing and supporting nascent efforts to develop UREs. Unlike the students in Dortch and Patel’s study [9], our findings suggest that UREs can help women and students of color feel like they can thrive when they are around racially diverse peers and faculty, especially when they share one or more background characteristics. Furthermore, the interview data underscored the significance of the temporal dimension in how students develop connections with faculty and peers, ultimately cultivating a stronger sense of belonging through prolonged participation in a URE. Many studies focus on students within a single cohort [13] or in shorter-term UREs [36]. Bowman and Holmes [13] found that URE participation was not related to first-year students’ satisfaction in college. However, we suggest that sense of belonging may be a more meaningful affective outcome than satisfaction and further suggest that it may take more than one year for a URE to lead to a stronger sense of belonging.

In prior work, members of the research team have outlined their approach to designing the URE described in this paper as well as training faculty mentors [20,39]. Members of the research team [47] have also shown that this specific URE’s mentoring approach is positively related to the classroom sense of community scale [46] and the community belonging scale [45]. Along with those prior studies, these new findings offer insights and potentially transferrable implications for university faculty and administrators who aim to prioritize supporting students’ sense of belonging as they develop as students and future researchers. Future research may examine other features of this URE, and other UREs in general, such as the importance of offering financial support to help students succeed and feel a sense of belonging. Researchers may also consider how UREs can foster a sense of “mattering”, which shifts from feeling as if one belongs to focusing on being intrinsically valued [53].

## 10. Conclusions

The nation’s public health and economic competitiveness depend on increasing the number and diversity of students who are preparing for research careers in STEM and the biomedical sciences. However, traditional academic departments and UREs can leave underrepresented students feeling isolated and anxious. This study offers important, novel insights that challenge traditional expectations that students need to change to fit into the culture of the sciences. Instead, through mixed-methods research, this study offers multiple examples of how a URE can foster sense of belonging through mentoring and embracing diversity and caring. As indicated by the quote in the title of this paper, the emotional support enhances—and is not tangential—to the research training itself.

## Figures and Tables

**Table 1.** Summary of sample aggregation.

		Class Standing at Panel Entry			
Year of Panel Entry	Freshman	Sophomore	Junior	Senior	Total

2020	27	33	75	84	219
2021	0	7	6	12	25
2022	0	0	10	15	25
2023	0	0	5	4	9
Total	27	40	96	115	278

**Table 2.** Demographic characteristics for survey sample.

Race or Ethnicity (White as Reference)	Percent

Asian	19%
Black	9%
Hispanic	53%
Other	6%
First-generation college student	67%
Woman (man as reference)	83%
Current undergrad (alumni as reference)	30%
Participated in URE	18%
Applied to graduate school	19%

**Table 3.** Descriptive statistics of survey sample.

	Mean	Std. Dev.	Min	Max

Sense of belonging (current undergraduates)	14.18	3.13	6	20
Sense of belonging (alumni)	13.85	3.79	2	20
Sense of belonging (pooled)	13.95	3.60	2	20
Age	22.26	3.97	19	51
Duration of commute (minutes)	28.83	23.68	0	100

**Table 4.** Summary of oral history participation.

	Number of Invited Narrators	Number of Participating Narrators	Number of URE Narrators

2020	45	28	14
2021	45	36	19
2022	37	36	20
2023	33	29	16

**Table 5.** Ordinary least squares regression results testing relationship between participating in undergraduate research experience program and sense of belonging.

	Current Undergraduates Only				Alumni Only				Pooled Sample			

	Coef.	Std. Err.	P > t	Beta	Coef.	Std. Err	P > t	Beta	Coef.	Std. Err.	P > t	Beta

**Year of Panel Entry (2020 as Reference)**												
2021	−1.83	1.84	0.33	−0.14	0.14	1.15	0.91	0.01	0.17	0.95	0.86	0.01
2022	1.22	1.59	0.45	0.14	−1.55	1.63	0.34	−0.07	−0.35	1.08	0.75	−0.02
2023	0.95	2.04	0.64	0.10					0.00	1.63	1.00	0.00
**Class Standing at Panel Entry (First-Year as Reference)**												
Sophomore	1.16	1.05	0.27	0.17	−3.98	2.61	0.13	−0.21	−0.57	1.06	0.59	−0.05
Junior	−1.05	1.44	0.47	−0.14	−1.41	2.29	0.54	−0.17	−0.47	1.13	0.68	−0.06
Senior	0.75	1.78	0.67	0.09	−0.49	2.28	0.83	−0.06	0.74	1.17	0.53	0.10
**Race or Ethnicity**												
Asian	−1.73	1.17	0.15	−0.23	0.40	0.88	0.65	0.04	−0.06	0.71	0.93	−0.01
Black	1.44	1.66	0.39	0.12	−1.53	1.19	0.20	−0.10	−0.78	0.97	0.42	−0.05
Hispanic	−0.39	1.07	0.71	−0.06	1.58	0.76	0.04	0.20	1.13	0.62	0.07	0.15
Other					0.74	1.15	0.52	0.05	0.34	1.08	0.75	0.02
Age	−0.03	0.20	0.87	−0.03	−0.13	0.08	0.10	−0.13	−0.10	0.07	0.14	−0.10
Duration of commute	0.01	0.02	0.48	0.10	−0.02	0.01	0.14	−0.11	−0.01	0.01	0.23	−0.08
First-generation college student	0.93	0.98	0.35	0.13	−0.81	0.74	0.27	−0.10	−0.40	0.59	0.50	−0.05
Woman (man as reference)	−0.63	1.02	0.54	−0.08	−0.44	0.80	0.59	−0.04	−0.40	0.65	0.53	−0.04
Current undergrad									0.57	0.82	0.48	0.07
Participated in URE	2.61	1.47	0.08	0.34	2.06	0.79	0.01	0.20	2.23	0.67	0.00	0.23

	*R^2^* = 0.30				*R^2^* = 0.15				*R^2^* = 0.12			
	*N* = 69				*N* = 172				*N* = 241			

Note: Following a listwise deletion approach, regression analyses were completed using complete cases.

**Table 6.** Logistic regression results testing relationship between sense of belonging and odds of applying to graduate school.

	Pooled Sample		

	OR	Std. Err.	P > t

**Year of Panel Entry (2020 as Reference)**			
2021	0.76	0.54	0.70
2022	2.16	1.63	0.31
2023	1.89	2.15	0.58
**Class Standing at Panel Entry (First-Year as Reference)**			
Sophomore	3.32	2.99	0.18
Junior	0.74	0.73	0.76
Senior	1.29	1.27	0.80
**Race or Ethnicity**			
Asian	1.44	0.74	0.47
Black	0.27	0.30	0.24
Hispanic	0.92	0.42	0.85
Other	0.73	0.57	0.69
Age	1.08	0.05	0.09
Duration of commute	1.01	0.01	0.46
First-generation college student	1.08	0.48	0.86
Woman (man as reference)	2.48	1.41	0.11
Current undergrad	0.51	0.34	0.32
Sense of belonging (standardized)	1.79	0.36	0.00
Participated in URE	1.31	0.60	0.56

	*Pseudo R^2^* = 0.11		
	*N* = 241		

**Table 7.** Table summarizing how themes were developed from categories.

Categories	Themes

1. Faculty Show Authentic Interest in Students	1. Faculty directly communicate to students whether they think they belong.
2. Personal Connections with Faculty	
3. Feeling Cared For	
4. There is a Culture of Diversity	2. By determining program composition, content, and activities, UREs can create smaller communities of belonging.
5. Groups Facilitate Involvement	
6. Feeling Similarity or Shared Backgrounds with Others	
7. People on Campus Are Friendly and Helpful	3. The university environment is congruent with positive involvement with faculty and peers through the URE.
8. People on Campus Are Relatable	
9. People are Welcoming and Approachable	
10. Connected through Regular Communication	

## Data Availability

The datasets used and/or analysed during the current study are available from the corresponding author on reasonable request.
